# Assessment of Diversity of Antimicrobial Resistance Phenotypes and Genotypes of *Mannheimia haemolytica* Isolates From Bovine Nasopharyngeal Swabs

**DOI:** 10.3389/fvets.2022.883389

**Published:** 2022-05-11

**Authors:** Hannah F. Carter, Robert W. Wills, Matthew A. Scott, Alexis C. Thompson, Randall S. Singer, John Dustin Loy, Brandi B. Karisch, William B. Epperson, Amelia R. Woolums

**Affiliations:** ^1^Department of Pathobiology and Population Medicine, College of Veterinary Medicine, Mississippi State University, Starkville, MS, United States; ^2^Department of Comparative Biomedical Sciences, College of Veterinary Medicine, Mississippi State University, Starkville, MS, United States; ^3^Veterinary Education, Research, and Outreach Center, Texas A&M University and West Texas A&M University, Canyon, TX, United States; ^4^Department of Veterinary and Biomedical Sciences, College of Veterinary Medicine, University of Minnesota, Saint Paul, MN, United States; ^5^School of Veterinary Medicine and Biomedical Sciences, University of Nebraska-Lincoln, Lincoln, NE, United States; ^6^Department of Animal and Dairy Science, College of Agriculture and Life Sciences, Mississippi State University, Starkville, MS, United States

**Keywords:** cattle, respiratory, bacteria, pasteurellaceae, resistance

## Abstract

The threat of bovine respiratory disease (BRD) for cattle operations is exacerbated by increasing prevalence of antimicrobial resistance (AMR) in *Mannheimia haemolytica*, a leading cause of BRD. Characterization of AMR in *M. haemolytica* by culture and susceptibility testing is complicated by uncertainty regarding the number of colonies that must be selected to accurately characterize AMR phenotypes (antibiograms) and genotypes in a culture. The study objective was to assess phenotypic and genotypic diversity of *M. haemolytica* isolates on nasopharyngeal swabs (NPS) from 28 cattle at risk for BRD or with BRD. NPS were swabbed onto five consecutive blood agar plates; after incubation up to 20 *M. haemolytica* colonies were selected per plate (up to 100 colonies per NPS). Phenotype was determined by measuring minimum inhibitory concentrations (MIC) for 11 antimicrobials and classifying isolates as resistant or not. Genotype was indirectly determined by matrix-assisted laser desorption/ionization time of flight mass spectroscopy (MALDI-TOF MS). NPS from 11 of 28 cattle yielded at least one *M. haemolytica* isolate; median (range) of isolates per NPS was 48 (1–94). NPS from seven cattle yielded one phenotype, 3 NPS yielded two, and 1 NPS yielded three; however, within a sample all phenotypic differences were due to only one MIC dilution. On each NPS all *M. haemolytica* isolated were the same genotype; genotype 1 was isolated from three NPS and genotype two was isolated from eight. Diversity of *M. haemolytica* on bovine NPS was limited, suggesting that selection of few colonies might adequately identify relevant phenotypes and genotypes.

## Introduction

Bovine respiratory disease (BRD), the leading cause of morbidity and mortality in U.S. beef cattle (1), poses a threat to cattle operations. The prevalence of antimicrobial resistance (AMR) appears to be increasing in *Mannheimia haemolytica* (*M. haemolytica*), a leading contributor to BRD (2–4); research is underway to determine the causes and impact of AMR in *M. haemolytica*. Characterization of the AMR phenotype by culture and antimicrobial susceptibility testing is complicated by uncertainty regarding the number of *M. haemolytica* colonies that must be selected to adequately characterize antimicrobial susceptibility phenotypes (antibiograms) and genotypes in a sample. While multiple colonies consistent with *M. haemolytica* may be present on a primary culture plate from a bovine sample, standard diagnostic methodology is to select one isolate for characterization. It may be that selection of multiple colonies is necessary to accurately identify important AMR isolates, but this could substantially amplify the cost of testing. As research is ongoing to characterize the extent and impact of AMR in the bacteria that contribute to BRD, it is important to clarify whether a single isolate from a respiratory sample adequately represents the characteristics of all isolates that can be identified in the same sample.

The number of colonies that must be isolated from a primary culture plate to accurately represent the diversity of isolates on the plate has been determined for other bacteria (5, 6), but to our knowledge this number has not been estimated for *M. haemolytica*. The study objective was to describe the phenotypic and genotypic diversity of up to 100 *M. haemolytica* isolates from individual bovine nasopharyngeal swabs (NPS) collected from live cattle at risk for BRD, or after treatment for BRD.

## Materials and Methods

### Animals

Subject cattle (*n* = 28) were a convenience sample of post-weaned mixed breed beef cattle of *Bos taurus* origin, weighing 180–270 kg, with an estimated age of 6 months to 1 year. The cattle were in different groups of recently purchased and comingled cattle from various auction markets. At the time of sampling the cattle had received zero to three treatments with an antimicrobial approved for treatment of BRD. The cattle were sampled either at a convenient time post arrival or when they were removed from their pen to be treated for BRD. Because the primary objective of the study was to describe the variability of *M. haemolytica* phenotypes and genotypes isolated from bovine NPS, a mix of both previously treated and untreated cattle was included so that it was possible to ascertain whether previous treatment was likely to impact variability. Sample collection for this study was approved by the Mississippi State University Institutional Animal Care and Use Committee (IACUC 17-330).

### Nasopharyngeal Swab (NPS) Collection and Culture

Double guarded swabs (#022964 MWI, Nampa, ID, USA) were used to sample the nasopharynx of cattle as previously described (7). One swab was collected from each nostril then the two swabs were placed together into transport media (Modified Amies Clear gel, SP130X, Starplex Scientific Inc. Etobicoke, Ontario, Canada) and transported back to the laboratory on ice for culture within 6 h of collection. Both swabs were streaked together on the first quadrant of 5 sequential plates containing tryptic soy agar + 5% sheep's blood (blood agar) plates. Five sequential plates were streaked in order to account for the possibility that overgrowth of contaminant bacteria might prevent identification of *M. haemolytica* on the first plate. For each of the 5 plates a new sterile loop was used to streak from the first quadrant to the remaining 3 quadrants. Plates were streaked and evaluated in a biosafety cabinet to prevent contamination. After streaking, plates were incubated at 37°C, 5% CO_2_ for 18–24 h, then colonies phenotypically consistent with *M. haemolytica* (round white/gray with a glossy edge and beta hemolysis) were collected, with one colony tested by the oxidase (slow +), indole (–), catalase (+), and KoH (+) tests to confirm identity. If there were fewer than 3 colonies on a plate the biochemical tests were not performed until the isolates on the primary plate were subcultured. Isolated colonies consistent with *M. haemolytica* were subcultured to a new plate; a maximum of 20 colonies were collected from each of the 5 plates, for up to 100 colonies per NPS. The subcultures were incubated at 37°C in 5% CO_2_ for 18–24 h, then for each subculture plate all bacteria were swabbed off and transferred to 1 ml of 50% glycerol in 1X phosphate buffered saline, and stored at −80°C.

### Broth Microdilution for Determination of Antimicrobial Susceptibility

**Figure 1:**
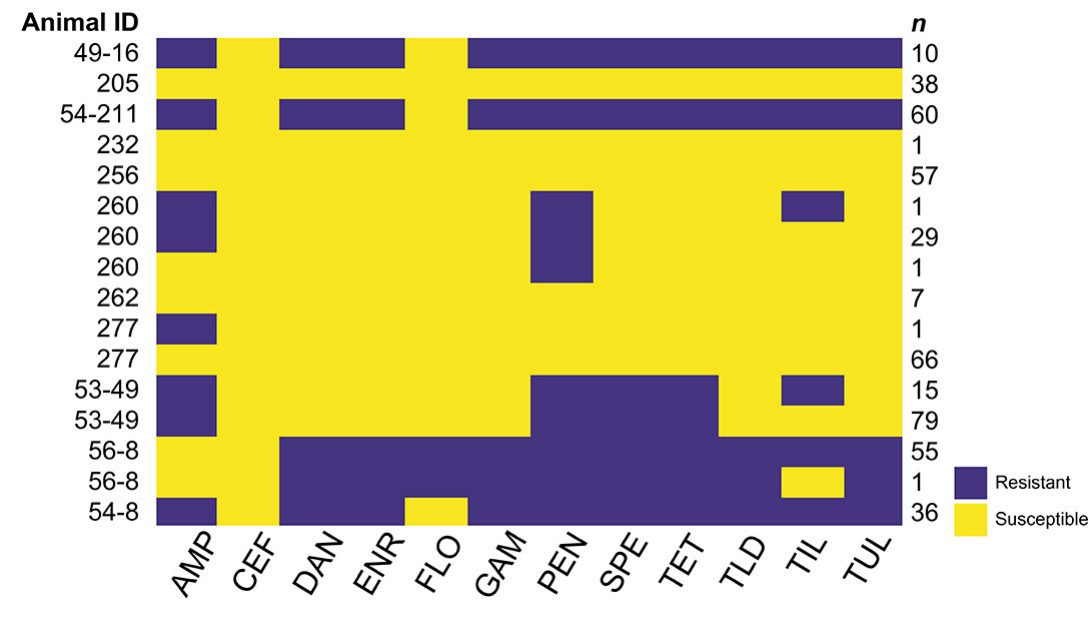
Antimicrobial resistance (AMR) phenotypes (antibiograms) of *M. haemolytica* isolates from nasopharyngeal swabs (NSP) collected from 11 cattle. The median number (range) of *M. haemolytica* isolates obtained from each animal was 48 (1–94). Antimicrobials: ampicillin (AMP), ceftiofur (CEF), danofloxacin (DAN), enrofloxacin (ENR), florfenicol (FLO), gamithromycin (GAM), penicillin (PEN), spectinomycin (SPE), tetracycline (TET), tildipirosin (TLD), tilmicosin (TIL), and tulathromycin (TUL). Yellow cells indicate that the isolates were not resistant to the antimicrobial indicated, while purple cells indicate resistance. While multiple AMR phenotypes were identified among *M. haemolytica* isolates from four cattle (260, 277, 53–49, and 56–8), the difference in phenotype was in all cases due to a difference of only a single dilution in the broth microdilution assay, which led to the isolate changing from not resistant to resistant for only one or two antimicrobials. The relevant minimum inhibitory concentration (MIC) data are presented in Supplementary Material 1.

Twelve to 14 months after the NPS were collected, isolates were removed from −80°C storage, transferred to ice, and immediately streaked onto blood agar plates. After 18–24 h of incubation at 37°C in 5% CO_2_ each isolate was tested to confirm genus and species using an automated system (Sensititre, ThermoFisher etc., plate YGNID), and the minimum inhibitory concentration (MIC) for 11 antimicrobials was determined by broth microdilution (Sensititre, ThermoFisher etc., plate YBOPO7F) at the Mississippi State University College of Veterinary Medicine Diagnostic Laboratory (MSU CVM DL). MICs were determined for ceftiofur, danofloxacin, enrofloxacin, florfenicol, gamithromycin, penicillin, spectinomycin, tetracycline, tildipirosin, tilmicosin, and tulathromycin; each isolate was identified as susceptible, intermediate, or resistant based on CLSI-defined breakpoints for *M. haemolytica* in bovine respiratory disease. For subsequent evaluation in this study, intermediate isolates were grouped with susceptible isolates, so that each isolate was defined as resistant or non-resistant. A figure representing the phenotypes represented by isolates (Figure 1) was constructed in R v4.0.4, using the Bioconductor package ComplexHeatmap v2.10.0 (8). Color scaling was performed with the R package viridis v0.6.2 (9) to allow ease of visual interpretation for individuals with color blindness.

### MALDI-TOF MS

For each isolate, the broth used for MIC determination was also used to inoculate a blood agar plate which was incubated for 18–24 h at 37°C in 5% CO_2_ for confirmation of identity by matrix-assisted laser desorption/ionization-time of flight mass spectrometry (MALDI-TOF MS). If a plate inoculated with broth used for antimicrobial susceptibility testing yielded no growth or contaminated growth, so that the isolate could not also be confirmed as *M. haemolytica* by MALDI-TOF MS, that isolate was omitted from the analysis. This led to exclusion of 9 of 57 isolates from animal 205, 10 of 51 isolates from animal 260, 3 of 73 isolates from animal 277, and 2 of 61 isolates from animal 256. For MALDI-TOF MS isolates were shipped by overnight mail to the University of Nebraska-Lincoln Veterinary Diagnostic Center (UNL VDC), where isolated colonies were prepared in duplicate according to manufacturer's recommended procedures for the direct smear method using a α-cyano-4-hydroxycinnamic acid matrix (Bruker Daltonics, Billerica, MA, USA) and subjected to automatic detection in positive linear mode between 2 kDA and 20 kDA m/z, with a laser frequency of 60 Hz using a Microflex LT MALDI-TOF mass spectrometer (Bruker Daltonics) calibrated for reference masses of 3,637–16,952 Da using the manufacturer's supplied bacterial test standard. Identifications were determined using commercial software (Bruker Biotyper, Bruker Daltonics) and the manufacturer's database (BDAL v10 containing 9,607 reference spectra) that has been supplemented with an in-house developed library with additional *Mannheimia* spp. reference spectra. Isolates were identified to the species level if match scores on at least one replicate were ≥ 2.2. The MALDI-TOF MS profile generated during identification was also used to assign each isolate to genotype 1 or 2 as previously described using Clinprotools 3.0 software (Bruker Daltonics) with quick classifier model, which was developed based on whole genome sequences of known genotype 1 and genotype 2 *M. haemolytica* isolates, of which there are >26,000 nucleotide polymorphisms that discriminate between the two genotypes. In addition to the classifier model, a manual review of raw mass spectrum peaks was also included to verify proper genotype classification (10). *Mannheimia haemolytica* genotypes 1 and 2 that are identified by this MALDI quick classifier model were previously described by Clawson et al. (11), with genotype 2 isolates primarily originating from the lungs of cattle with clinical or pathological signs of respiratory disease, and typically harboring integrative conjugative elements (ICE) conferring multi-drug antimicrobial resistance, and genotype 1 isolates originating from the upper respiratory tract of cattle with no signs of disease, and typically not including ICE. For strains where there is genomic information available, genotype 1 strains are likely serotype 2 based on molecular analysis and genotype 2 strains are either serotype 1 or serotype 6 based on the same analysis, suggesting a strong relationship between serotype and genotype (12).

## Results

### Animals and Bacterial Culture Results

Nasopharyngeal swabs were collected from cattle in four different groups (A–D) between March - May 2018 or in May 2019. Seven cattle were sampled in group A, six cattle were sampled in group B, five cattle were sampled in group C, and 10 cattle were sampled in group D. The seven cattle in group A were each sampled on two different occasions 15 days apart, but *M. haemolytica* was isolated from each animal only once, or not at all (four cattle positive when sampled the first time, two cattle positive when sampled the second time, and one animal negative at both sampling times). Cattle in groups B–D were only sampled once. Nasopharyngeal swabs from 11 of the 28 cattle yielded at least one *M. haemolytica* isolate. Details regarding the 11 cattle from which *M. haemolytica* were isolated are presented in Table 1. Two cattle (205 and 260) had not been treated with antimicrobials at the time of sampling, but they were treated for BRD based on clinical signs 2 days after they were sampled; none of the other cattle in that group (group A) were ever treated for BRD in the approximately 90-day period during which they were monitored. For each animal the median number (range) of *M. haemolytica* isolates obtained from the first plate streaked was 8 (0 −17), and the median number (range) of isolates from all 5 plates streaked was 48 (1–94) (Table 2). *M. haemolytica* was not identified on the first plate streaked for two of the cattle, and for one of these two cattle (232), only one *M. haemolytica* isolate was identified, on plate 5.

**Table 1:** Information regarding cattle from which *Mannheimia haemolytica* (*M. haemolytica*) was isolated from nasopharyngeal swabs (NPS), the number of AMR phenotypes (antibiograms) of M. haemolytica identified among all isolates, and the genotype of *M. haemolytica* isolated based on MALDI-TOF MS.

**Animal ID**	**Housing group**	**Clinical signs of BRD when sampled**	**Antimicrobials received before sampling (days before)**	**Number of AMR phenotypes**	**Genotype**
205	A	no	none	1	1
232	A	no	none	1	1
260	A	no	none	3	2
277	A	no	none	2	2
256	A	no	none	1	1
262	A	no	none	1	2
56–8	B	yes	CEF (34) TUL (19) FLO (9)	2	2
53–49	C	yes	CEF (8)	2	2
49–16	D	yes	CEF (35) TUL (15) FLO (12)	1	2
54–8	D	yes	CEF (21) TUL (3)	1	2
54–211	D	yes	CEF (14) TUL (7)	1	2

*Cattle in the same housing group were housed and managed together. Although multiple phenotypes of M. haemolytica were isolated from some cattle, all isolates identified from each animal were a single genotype, either genotype 1 or genotype 2. Antimicrobials: CEF, ceftiofur, TUL, tulathromycin, FLO, florfenicol*.

**Table 2:** Number of *Mannheimia haemolytica* (*M. haemolytica*) isolates recovered from nasopharyngeal swabs (NPS) from 11 cattle.

	**Animal ID**										
**Plate number**	**205**	**232**	**260**	**277**	**256**	**262**	**56–8**	**53–49**	**49–16**	**54–8**	**54–211**
1	8	0	4	14	13	6	8	17	0	10	9
2	11	0	7	15	5	0	19	19	4	11	13
3	7	0	10	15	11	0	12	18	3	8	12
4	16	0	9	14	15	0	6	20	2	2	13
5	6	1	11	12	15	1	11	20	1	5	13
Total *M. haemolytica* Isolates	48	1	41	70	59	7	56	94	10	36	60
Number of AMR phenotypes	1	1	3	2	1	1	2	2	1	1	1
*M. haemolytica* genotype	1	1	2	2	1	2	2	2	2	2	2

*Two NPS (one from each nostril) were together streaked onto the first quadrant of 5 consecutive blood agar plates (plate numbers 1 through 5), then the remaining three quadrants of each plate were streaked with a new sterile loop. Each individual colony (up to 20 for each plate) was subcultured once for determination of minimum inhibitory concentrations for 11 antimicrobials to determine AMR phenotype, and for MALDI-TOF MS to determine genotype*.

#### Phenotypes of Isolates

The AMR phenotype, or antibiogram, of each *M. haemolytica* isolate from each NPS was defined by the antimicrobial susceptibility to each antimicrobial tested in the broth microdilution assay. Isolates were defined as resistant or not resistant, with isolates having MIC in the intermediate range included with isolates in the sensitive range. The AMR phenotypes identified in the *M. haemolytica* isolates from each of the 11 animals described in Table 2 are presented in Figure 1, and the MIC data for all antimicrobials for each isolate are presented in Supplementary Material 1. Nasopharyngeal swabs from 7 cattle yielded *M. haemolytica* with only one phenotype, NPS from 3 cattle yielded M. haemolytica with 2 phenotypes, and an NPS from one animal yielded *M. haemolytica* with 3 phenotypes. Differences in MIC among isolates from an individual animal that led to changes in phenotype were found for penicillin, tetracycline, or tilmicosin (Supplementary Material 1). However, when more than one phenotype was identified among the *M. haemolytica* isolates from the NPS from an individual animal, the difference between the phenotypes was always due to a difference of only one dilution near the breakpoint, which led to some isolates from an animal being defined as sensitive while others were identified as resistant. Since a difference of one dilution can be interpreted to be within the error of the broth microdilution assay, in this study all *M. haemolytica* isolates from the same NPS had essentially the same AMR phenotype.

#### Genotypes of Isolates

All isolates confirmed to be *M. haemolytica* by both Sensititre and by MALDI-TOF MS were assigned to genotype 1 or 2 based on the MALDI-TOF MS profile as described (10). All isolates obtained from the NPS from an individual animal were the same genotype. The isolates from NPS from 3 cattle were genotype 1, and the isolates from NPS from 7 cattle were genotype 2 (Tables 1, 2 and Supplementary Material 1).

## Discussion

Planning research to evaluate AMR in BRD leads to a recurring question: “How many *M. haemolytica* colonies do we need to select from a primary culture plate to have confidence that we have identified all the relevant isolates?”. This research was undertaken to address this question. In work evaluating gamithromycin susceptibility of *M. haemolytica* isolates from bovine NPS or bronchoalveolar lavage fluid samples, Capik et al. (13) found that, when up to 12 *M. haemolytica* colonies were selected from primary plates, a mixture of sensitive and resistant isolates was sometimes found. However, that report did not provide exact numbers of sensitive and resistant isolates identified in individual samples, and it did not provide information for antimicrobials other than gamithromycin. To our knowledge no other research has described the number of different AMR phenotypes that can be identified in *M. haemolytica* isolated from the same bovine respiratory sample.

The number of colonies that need to be selected from a primary culture plate to accurately represent the diversity of isolates on the plate has been estimated for other bacterial pathogens. Singer et al. (5) developed a model to predict the number of isolates that need to be tested to determine with a high level of confidence the diversity of *Escherichia coli* (*E. coli*) isolates from cases of avian cellulitis. In this work the *E. coli* phenotype was defined by DNA pulsed field gel electrophoresis, and the model developed indicated that if 3 randomly selected colonies were phenotypically identical, the probability was 98.8% that only one phenotype was present on the plate. In other research, Döpfer et al. (6) developed a model to predict the number of isolates that need to be selected to identify all phenotypes of *E. coli, Listeria monocytogenes, Klebsiella pneumoniae*, or *Streptococcus uberis* on a culture plate, with phenotype defined by ribotyping, pulsed-field gel electrophoresis, or PCR-based strain-typing methods. The model of Döpfer et al. indicated that, for the bacteria evaluated, between 2 and 20 isolates needed to be selected and characterized to identify all phenotypes in the sample with 95% certainty. While the models of Singer et al. or Döpfer et al. should be applicable to *M. haemolytica*, they are based on Bayesian inferences that require an estimate of the number of different phenotypes (“prior information” or “prior probability”) expected. The work presented here was undertaken to obtain this prior information, so that such models could be used to estimate the number of *M. haemolytica* colonies that need to be selected from a plate to provide a high level of confidence regarding the number of AMR phenotypes on the plate. However, the results indicated a surprising uniformity of phenotype, with essentially no diversity, and therefore the data did not support the use of a model to predict diversity. Since the phenotypes and genotypes of isolates from a sample were quite uniform, it appears that selecting one isolate may indeed adequately represent the characteristics of *M. haemolytica* isolates from bovine NPS. Put another way, the data suggest that a very large number of isolates would need to be tested to identify rare diverse isolates, which may not be feasible in terms of logistics or cost.

In addition to uniformity of AMR phenotype, the samples evaluated here revealed uniformity of *M. haemolytica* genotype, which was identified by the MALDI-TOF MS profile (10). This finding is similar to the results reported by Capik et al. (13), who showed that, when up to 12 *M. haemolytica* isolates were selected from culture plates from individual bovine NPS or bronchoalveolar lavage fluid samples, DNA sequencing and construction of phylogenetic trees revealed more than one genotype in only one of 12 samples described. In other work by the same group, characterization of multiple *M. haemolytica* isolates from the same bovine NPS culture showed little diversity as defined by DNA pulsed-field gel electrophoresis (14). Similarly, characterization of plasmid types from up to 8 *M. haemolytica* isolates from nasal swabs from feedlot cattle with or without BRD revealed that fewer than 10% of samples yielded more than one plasmid type (15), and evaluation of at least three *M. haemolytica* isolates from nasopharyngeal swabs from feedlot cattle demonstrated that isolates were in most cases identical based on pulse field gel electrophoresis (16). It should be noted that none of the genotyping methods used to characterize diversity of *M. haemolytica* isolates obtained from a single bovine respiratory sample, including our use of the MALDI quick classifier model to identify genotypes 1 and 2 described by Clawson et al. (11), provide the same resolution as whole genome sequencing. The genotyping approach used in this report is more similar to serotyping, where strains are classified broadly based on >26,000 nucleotide polymorphisms and have associations with capsular genes. Therefore, some differences among these apparently uniform isolates may have been present that would have been identified by whole genome sequencing or typing methods with higher resolution.

It has been reported (11) that genotype 2 *M. haemolytica* are most often isolated from cattle with clinical signs of BRD, while genotype 1 isolates are most often isolated from cattle that are clinically healthy. The results of the present study were generally consistent with this pattern, in that NPS from five of five cattle sampled at the time of BRD treatment yielded a genotype 2 *M. haemolytica*. Of the six cattle sampled when not showing signs of BRD, three cattle yielded a genotype 1 *M. haemolytica*, while genotype 2 *M. haemolytica* was isolated from the other three. However, one of the three “non-BRD” cattle with a genotype 2 *M. haemolytica* (animal 260) was treated for BRD 2 days after it was sampled.

In this study, NPS were streaked onto the first quadrant of five consecutive plates, in order to increase the likelihood of finding diverse *M. haemolytica* isolates that might have been overgrown by other bacteria on the first plate. This technique is not a standard practice in diagnostic laboratories, but given the fact that *M. haemolytica* was not identified on plate 1, but was identified on subsequent plates for two of the 11 cattle from which *M. haemolytica* was identified, the approach may be warranted in research. The lack of diversity across plates for each sample suggests that, once *M. haemolytica* is identified on one plate, the phenotype and genotype are likely to be similar to those identified on another plate from the same sample.

Limitations of this study include the relatively small number of cattle and cattle groups sampled, and the fact that sampled cattle came from a relatively limited geographic region. Given the lack of diversity found in this study, and the cost of characterizing large numbers of isolates from a single sample, it may be difficult to justify the cost to repeat this research with a larger number of cattle or groups. However, it is important to note that the results reported here may not be representative for other types of respiratory samples (e.g. nasal swabs or bronchoalveolar lavage samples), or for samples from other types of cattle, such as dairy calves, or for other BRD agents, such as *Pasteurella multocida* or *Histophilus somni*. Confirmation of the diversity of respiratory isolates as related to these other variables will require additional research.

## Data Availability Statement

The original contributions presented in the study are included in the article/Supplementary Material, further inquiries can be directed to the corresponding author.

## Ethics Statement

The animal study was reviewed and approved by Mississippi State University Institutional Animal Care and Use Committee.

## Author Contributions

AW, RW, and HC: Experimental design. HC, JL, and WE: Data collection. RW, MS, AT, and RS: Analysis of data. BK and WE: Animal maintenance and supervision of sample collection. All authors contributed to review and editing of the final manuscript.

## Funding

This research was supported by United States Department of Agriculture Section 1433 Formula Funds, and the Mississippi State University College of Veterinary Medicine Summer Research Experience Program (NIH T35OD010432).

## Conflict of Interest

The authors declare that the research was conducted in the absence of any commercial or financial relationships that could be construed as a potential conflict of interest.

## Publisher's Note

All claims expressed in this article are solely those of the authors and do not necessarily represent those of their affiliated organizations, or those of the publisher, the editors and the reviewers. Any product that may be evaluated in this article, or claim that may be made by its manufacturer, is not guaranteed or endorsed by the publisher.
